# The Extent of Evidence Supporting the Effectiveness of Extended Reality Telerehabilitation on Different Qualitative and Quantitative Outcomes in Stroke Survivors: A Systematic Review

**DOI:** 10.3390/ijerph20176630

**Published:** 2023-08-23

**Authors:** Hatem Lazem, Abi Hall, Yasmine Gomaa, Maedeh Mansoubi, Sallie Lamb, Helen Dawes

**Affiliations:** 1 Medical School, Faculty of Health and Life Sciences, University of Exeter, Exeter EX1 2LP, UK; a.hall4@exeter.ac.uk (A.H.); m.mansoubi@exeter.ac.uk (M.M.); s.e.lamb@exeter.ac.uk (S.L.); h.dawes@exeter.ac.uk (H.D.); 2 Basic Science Department, Faculty of Physical Therapy, Cairo University, Cairo 12613, Egypt; 3 Faculty of Physical Therapy, Kafr Elsheikh University, Kafr Elsheikh 6860404, Egypt; yasmeen_gomaa@pt.kfs.edu.eg

**Keywords:** stroke, telerehabilitation, extended reality, virtual reality, augmented reality, motor, qualitative

## Abstract

*Objective*: To present the extent of evidence concerning the effectiveness of extended reality telerehabilitation and patients’ experiences of using different types of virtual reality exercises at home. *Methods*: We included studies on virtual reality and augmented reality telerehabilitation published in English. Systematic searches were undertaken in PubMed, Web of Sciences, Medline, Embase, CINAHL, and PEDro, with no date limitations. We included only RCTs and qualitative studies exploring patients’ experiences. Methodological quality was assessed using the Cochrane Risk of Bias assessment tool for quantitative papers and the CASP scale for qualitative studies. All results are presented narratively. *Results*: Thirteen studies, nine quantitative and four qualitative, were included, with one qualitative and seven quantitative having a high risk of bias. All studies reported that extended reality-based telerehabilitation may be effective compared to conventional exercises or other extended reality exercises. Seven quantitative studies focused on upper limb function. Qualitative papers suggested that VR exercises were perceived as feasible by patients. *Conclusions*: The literature suggests VR home exercises are feasible and potentially effective for patients after a stroke in the upper limb. Further high-quality studies are needed to examine the effectiveness of XR exercises early adoption on different qualitative and quantitative outcomes. Registration number: (CRD42022384356).

## 1. Introduction

Globally, stroke is the second-leading cause of death and the third-leading cause of disability [1]. It is well known that stroke survivors often suffer from several impairments and disabilities in motor and sensory functions, speech capacity, cognitive ability, and psychosocial difficulties [2]. The functional decline experienced by stroke survivors is commonly associated with increased dependence on daily activities and ultimately affects motivation levels, self-efficacy, and quality of life [3,4]. The physiotherapy service continues to play an important role in the interdisciplinary rehabilitation process, which includes occupational therapy, speech therapy, neuropsychology, and psychology, all of which contribute to the rehabilitation of post-stroke impairments, improving independence, and reducing disability [5]. However, there is a global shortage of rehabilitation services [6].

Telerehabilitation (TR) is the use of telecommunications technology for providing a variety of rehabilitation services [7]. Through TR, healthcare professionals can monitor the patient remotely and consult with the patient via teleconference without the need for the patient to be physically present. This can improve the patient’s ability to perform home exercises and reduce the need for clinic attendance [7,8,9,10]. Telerehabilitation may save time and money, especially for patients who live in distant areas with limited access to rehabilitation services [11,12].

Telerehabilitation can be delivered with similar principles to face-to-face rehabilitation, including goal setting, education, and social support for patients/families/careers [13,14,15,16,17].

A state of extended reality (XR) can be described as a term that encompasses three main technologies, including virtual reality, augmented reality, and mixed reality [18,19]. Virtual reality (VR) is increasingly being recognized as a valuable tool in neurorehabilitation and may be a useful adjunct to telerehabilitation [17,20,21,22]. VR may enable the provision of more intensive training for functional activities in conjunction with traditional therapies [22,23]. A virtual reality exercise is considered an innovative, non-invasive tool for giving patients a simulated experience with varying degrees of immersion and interaction with a virtual world [17]. Through the use of a joystick, mouse, sensors, camera, haptic device, or other gear, one can interact with a virtual environment [24,25]. It presents the opportunity for motor learning by testing the user’s ability to solve problems and master real-world skills in a virtual environment by giving feedback [26].

VR could be classified into three categories based on the degree of immersion: non-immersive, semi-immersive, and fully immersive [27,28]. In non-immersive VR, the patient uses the environment without fully immersing themselves, such as when playing a video game or performing physical activities using computer games [29]. Semi-immersive VR involves some degree of immersion but keeps the patient aware of what is happening outside of the virtual world [30]. The fully immersive VR experience allows the patient to be completely immersed in the virtual world and isolated from it by wearing a head-mounted display (HMD) or VR glasses [30,31]. The concept of augmented reality involves adding some virtual elements to the real world rather than immersing the user in a virtual environment [28].

Stroke patients, who typically have high rates of co-morbidity (for example, hypertension and diabetes mellitus), have experienced reduced and lower-quality healthcare services during the pandemic period of COVID-19, from delayed acute medical responses to delayed, canceled, or reduced rehabilitation appointments, especially for people who live in remote areas. Consequentially, there was a trend toward searching for a new tool of rehabilitation to overcome such risks and barriers [32,33,34]. Telerehabilitation using different models of extended reality provides a good solution to the need for a regular practice that helps patients obtain their rehabilitation services either with the supervision (synchronous) [35] or without the supervision (asynchronous) [36] of their therapists.

However, there is a gap in identifying the evidence for using XR in telerehabilitation of different motor outcomes, where completion of intervention components is described, and no systematic reviews have investigated the patient experience, facilitators, and barriers of using different types of extended reality exercises at home after stroke [9,10,37].

Therefore, our aim is to present the extent of the evidence concerning the effectiveness of extended reality telerehabilitation on motor outcomes and patients’ experiences when using different types of extended reality exercises at home after a stroke.

## 2. Materials and Methods

### 2.1. Protocol and Registration

The current review was conducted following the preferred reporting items for systematic reviews (PRISMA) statement [38]. The protocol was registered with PROSPERO under registration number: CRD42022384356.

### 2.2. Study Selection

This study has a mixed design that incorporates both qualitative and quantitative studies. As a part of the qualitative component of the review, we considered interventions, case studies, and mixed methods studies with relevant information regarding patients’ experiences and opinions regarding XR telerehabilitation at home. The quantitative component included all relevant randomized controlled trials (RCTs) that included extended reality (virtual reality or augmented reality) telerehabilitation at home as an experimental group and conventional physical therapy, usual care, or no care as a control group. Non-RCTs, pilot trials, and feasibility trials were excluded from the quantitative component. We included studies if they fulfilled the following criteria: Population: adults with stroke (≥18) of any cause (ischemic or hemorrhagic) at any stage after the lesion (acute–subacute–chronic), and with or without co-morbid conditions. Intervention: studies with any comparator that included any type of extended reality training as a home-based telerehabilitation tool (non-immersive, semi-immersive, fully immersive, augmented reality, and mixed reality) and excluded any studies that used extended reality in the assessment of stroke patients (for non-rehabilitation purposes). Outcomes: the quantitative component of this review considered studies that focus on the following primary outcomes: upper limb function, lower limb function, balance, ADL, mobility, trunk control, gait, and falls. The qualitative part of the review focused on including studies that focus on the following outcomes: patients’ views, experiences (opinions), needs, and expectations. Context: extended reality telerehabilitation exercises that were carried out at the patient’s home.

### 2.3. Search Strategy and Data Sources

Electronic and hand searches were conducted, in addition to searching through reference lists of relevant articles. The electronic databases included were PubMed, Web of Science (WoS), CINAHL, PEDro, and Medline. The search was limited to studies published in English from inception until 15 January. The search was performed by two authors (HL and AH; Tables S1 and S2).

### 2.4. Reviewing Procedures and Study Selection

**Figure 1:**
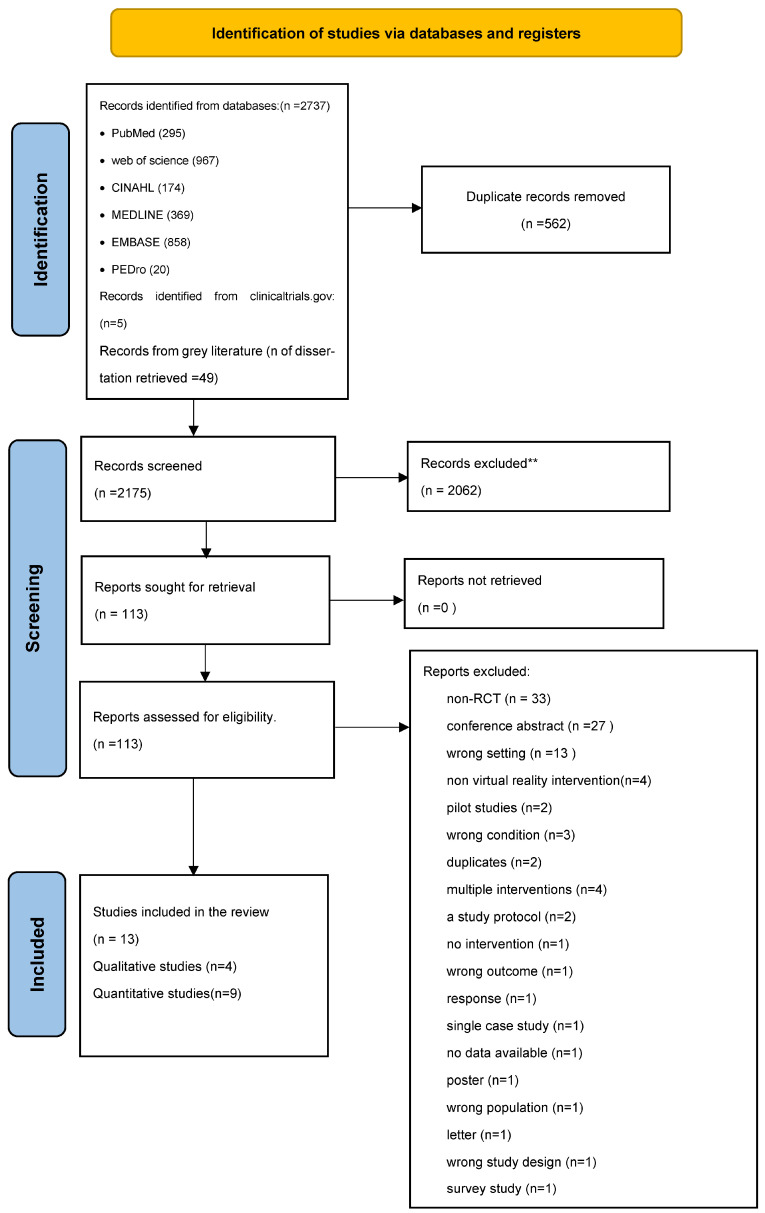
PRISMA flowchart for identifying studies for systematic review. ** Records that excluded after title and abstract screening.

Four researchers (HL–AH–YG–MM) performed the screening manually and independently. The first step included screening the titles and abstracts of all references retrieved from selected databases (HL–AH–MM) to identify the studies that met the inclusion criteria. Then, full-text screening was undertaken according to the predefined inclusion and exclusion criteria, and a decision to include or exclude the study was made by (HL), (AH), and (YG). A fourth reviewer (HD) resolved any disagreement at any step. All studies that did not meet the eligibility criteria were excluded. Reasons for exclusion are mentioned in the PRISMA chart. Studies that might appear to meet the inclusion criteria but were excluded after full-text screening can be found in the supplementary documents (Figure 1).

### 2.5. Data Extraction

Three authors (HL–AH–YG) extracted the data independently and in duplicate into a predetermined data extraction reporting Excel sheet. The following data was extracted from both qualitative and quantitative studies: author, year of publication, title, country, objectives, design, population characteristics (sample size, age, stage of stroke, time from stroke, multimorbid conditions, and scales used in baseline assessment), control and XR intervention details (type of intervention, dose, monitoring, setting, target region of rehabilitation, mode of delivery of intervention, and devices used to deliver the exercises), outcomes, and adverse effects. For the qualitative papers, we extracted more details regarding the underlying theory of the methods and methods of data collection and analysis.

### 2.6. Methodological Quality

We assessed RCT quality using Cochrane’s collaboration tool for assessing the risk of bias [39]. The CASP critical appraisal tool has been used for assessing qualitative studies [40]. Two reviewers (HL–YG) independently assessed the ROB of the quantitative studies, and two reviewers (HL–AH) independently assessed the ROB of the qualitative studies. A third author (HD) was contacted to resolve any discrepancies.

### 2.7. Data Synthesis and Analysis

All the quantitative studies included used heterogeneous types of XR interventions to address different motor outcomes. All but one study presented small (n < 200) sample sizes, and therefore effect sizes are presented but not further analyzed. Effect sizes were calculated using the formulas described by Lipsey and Wilson [41] (Table S3). Therefore, it was not possible to pool results using statistical meta-analysis, and all results were presented in narrative synthesis form.

## 3. Results

As illustrated in the PRISMA flowchart, 2737 references were identified. After the removal of 562 duplicates, 2175 records remained for the title and abstract screening phase. We excluded 2062 abstracts as they did not meet the inclusion criteria. Finally, we screened 113 full-text articles. Of these, we identified thirteen eligible papers: four qualitative and nine quantitative papers (Figure 1).

### 3.1. Risk of Bias Assessment

**Figure 2:**
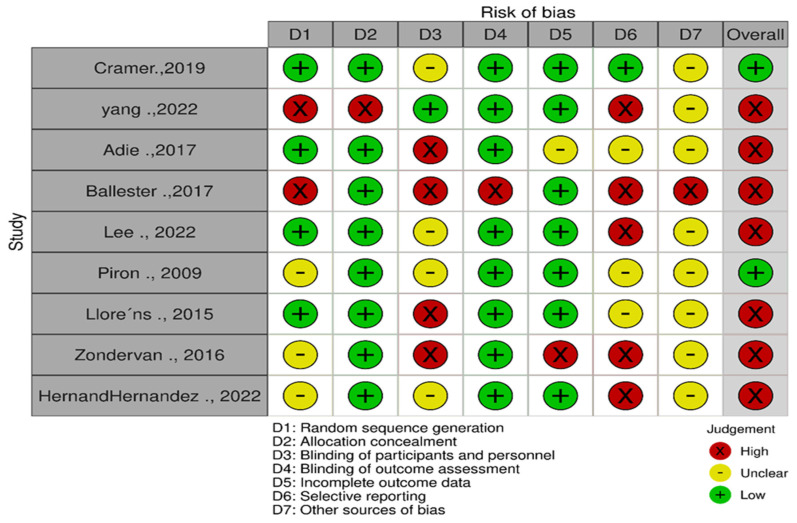
Risk of bias assessment graph for the quantitative studies (traffic plot) [35,36,42,43,44,45,46,47,48].

Using the Cochrane Collaboration tool, only two out of nine quantitative studies showed a low risk of bias in their overall score [42,43], while seven of the studies showed an overall high risk of bias [35,36,44,45,46,47,48] (Figure 2).

Regarding the qualitative studies, the CASP critical appraisal tool was used to assess the risk of bias. Overall, one paper was rated as low quality, while three papers were rated as moderate quality (Table 1).

### 3.2. Quantitative Studies

#### 3.2.1. Characteristics of Included Studies

The quantitative studies are outlined in Table S5. The nine RCTs were published between 2009 and These studies were conducted in various countries, including the UK [44], the USA [42,47], Spain [36,45], Hong Kong [35], Korea [46], Italy [43], and Canada [48].

#### 3.2.2. Participants

The mean age of the participants ranged from 55.4 to 68 years. The sample size ranged from 17 to 235 participants, with most strokes being hemorrhagic. Some patients had multimorbid conditions such as hypertension, diabetes mellitus, atrial fibrillation, hypercholesterolemia, and respiratory diseases [42,44]. All stroke survivors had mild to moderate arm impairments [43,48], mild spasticity without any severe contractures [35], and mild to moderate muscle weakness [35,44,45]. All participants had no cognitive or vision deficits that could affect their perception of the exercises. The upper extremity was the most common target region among all the studies (6/9), whereas (3/9) [35,36,46] focused on lower extremity function and balance outcomes.

#### 3.2.3. Types of Exercises and Mode of Delivery

Telerehabilitation exercises found in the eligible studies included non-immersive VR, semi-immersive VR, and augmented reality (AR) (Table S4).

#### 3.2.4. Dose of Exercises

XR intervention frequency varied in range from two sessions per week to daily sessions, and session duration ranged from 20 min to 120 min per session in total, with the treatment period ranging from three weeks to eight weeks.

#### 3.2.5. Tracking of the Treatment Plan

In most of the studies (5/9), a member of the research team revised the treatment plan so that it was possible to progress the exercises accordingly to ensure challenge for patients, as well as to encourage and motivate patients to complete their exercises or to report any adverse effects that may occur [35,42,43,46,48]. No study reported any data regarding patient adherence and compliance with the rehabilitation program.

#### 3.2.6. Devices Used to Deliver TR Exercises

TR exercises were delivered by a variety of elements, such as computers with monitors, an eye movement controller, a joystick, a Logitech trackpad, a Microsoft Kinect V2 RGB-D camera, software platforms, and gloves equipped with bend sensors that could be controlled remotely through the internet.

#### 3.2.7. Main Findings of Motor Outcomes

Two studies out of nine reported a non-serious adverse effect in the form of arm pain, general fatigue after the session, and dizziness. However, (1/9) studies reported serious adverse effects, all unrelated to the study [42]. Overall, only one study was powered (sample size > 200) to detect the change in the effect [44]. Most of the studies did not measure the compliance and adherence of the patients to their exercise program, which could influence the interpretation of the results.

##### Extended Reality Telerehabilitation Compared to In-Clinic Rehabilitation

There were four quantitative studies (4/9) that compared extended reality-based telerehabilitation exercises with in-clinic exercise therapy. In comparison to in-clinic rehabilitation, there was a significant improvement in upper limb function measured by Fugl–Meyer assessment upper extremity (FMA-UE) after using virtual reality telerehabilitation exercises at home, and this improvement was superior to in-clinic graded functional upper limb and postural control exercises [43], but this effect was not superior to in-clinic strengthening, stretching, active range of motion exercises when measured by FMA-UE, box and blocks test (BBT), and stroke impact scale (SIS) [35,42]. There was a significant improvement in lower limb function using augmented reality telerehabilitation lower limb exercises measured by Fugl–Meyer assessment lower extremity (FMA-LE), with no significant difference in comparison to the same augmented reality in-clinic training [35]. Regarding gait and balance outcomes, one study reported significant improvement in balance and gait, but this improvement was not superior to similar in-clinic virtual reality exercises when measured by the balance Berg scale (BBS), the performance-oriented mobility assessment balance subscale (POMA-B), or the performance-oriented mobility assessment gait subscale (POMA-G) [36]. However, another study showed a non-significant improvement in balance and gait domains, assessed by BBS and functional ambulation category (FAC), after using augmented reality exercises at home compared to a significant improvement after using the same exercises at the clinic [35] (Tables S5–S7).

##### Extended Reality Telerehabilitation Compared to Home Rehabilitation

There were five quantitative studies (5/9) that compared extended reality-based telerehabilitation exercises with other home-based exercise therapies. Two studies reported a significant improvement after using the Jintroux system and Nintendo sports games; this effect was similar to the effect of graded repetitive arm supplementary program (GRASP) upper limb exercises in one study, measured by action research arm test (ARAT) [44], but greater than GRASP upper limb exercises in another study that was measured by FMA-UE [48]. Regarding hand function, there was no significant improvement in hand function using semi-immersive virtual reality exercises combined with sensor gloves or music gloves among all outcome measures (FMA-UL, ARAT, nine-hole pig test, BBT, motor activity log) [45,47], except for the Chedoke arm and hand activity inventory (CAHAI) outcome measure, which reported a significant improvement in the experimental group but this improvement did not meet the minimal detectable change [45]. Only one study has reported an improvement in balance outcome and a decrease in risk of falls after using augmented reality exercises in the form of mobility, endurance, speed, and balance exercises, measured by time up and go (TUG), POMA, BBS, and falls efficacy scale-international (FES-I) [46] (Tables S5–S7).

### 3.3. Qualitative Studies

Results are summarized below according to characteristics of included studies, participants, and main findings using thematic synthesis Table S8.

#### 3.3.1. Characteristics of Included Studies

The qualitative studies are outlined in Table S5. The four qualitative studies were published between 2014 and These studies were conducted in the UK [50,51], the USA [52], and Canada [49].

#### 3.3.2. Participants

Participants’ mean values of age ranged between 58.8 and 70.61 years, with sample sizes ranging from three to eighteen participants with sub-acute to chronic stages of stroke. Among all studies, only two reported an underlying theory in the design: the unified theory of acceptance and use of technology (UTAUT) and the self-determination theory (SDT).

#### 3.3.3. Main Findings

**Figure 3:**
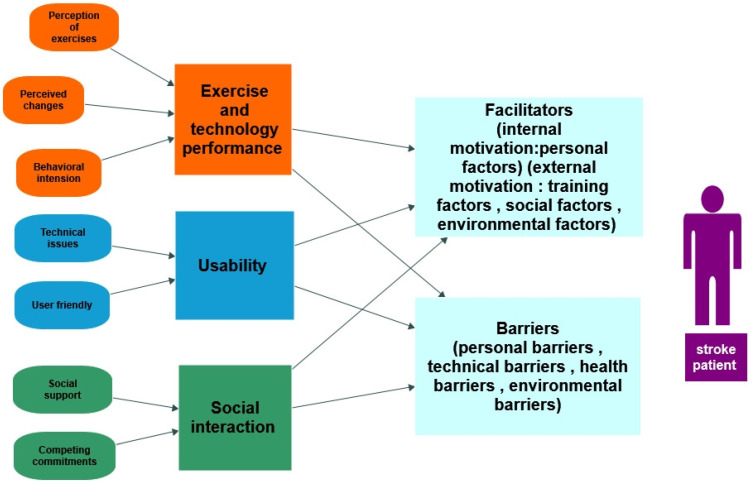
Summary of the results of the qualitative studies. This figure illustrates 3 main factors that may affect stroke patients’ experiences; (1) exercises and technology performance, which can be affected by the perception of exercises, perceived changes, and behavioral intentions of the patients; (2) usability, which can be affected by the technical issues of the VR platforms and the user experience; and (3) social interaction, which can be affected by the amount of social support and commitments of the patients. These factors can all affect VR exercises at home, whether as facilitators or barriers.

All studies have investigated patients’ experiences with non-immersive virtual reality telerehabilitation exercises in the form of Jintronix exergames, Wii games, virtual games using gloves, and gamified functional exercises targeting upper extremity and hand rehabilitation. Results could be categorized into five main overriding themes: exercises and technology performance, usability, social interaction, facilitators, and barriers (Figure 3).

##### Exercises and Technology Performance

Results suggest that patients had different perceptions regarding using gamified virtual reality exercises at home. Some patients thought that it was just an instrument of play and not tailored specifically to their condition [49], while others believed in its therapeutic nature and enjoyed using these exercises, as mentioned by one patient: ‘*Because it helps well, it helps you a lot in your movement. First and foremost, with the position, you know, then you enjoy the games*’ [49,51].

Stroke patients have reported some perceived changes regarding improvement in physical abilities and performing their ADL activities. As one patient said, “*my arm started getting a little stronger I could reach more you know in and I practiced I started reaching for the refrigerator with my right hand and door knobs*.” [50,52]; improvement in mental well-being in the form of enhancing their memory, feelings, and abilities to learn more about their condition, as mentioned by one participant: ‘*I was feeling emotional after the…stroke, um, because I wasn’t well enough to do anything round the house… but that [the WiiTM] just perked me up and made me feel useful*’ [50,52]. Improvement in social and emotional well-being, so patients become more socially connected through videoconferencing with clinicians [49].

In addition, some patients reported their behavioral intention to use the virtual reality exercise system in the future, but with more levels of difficulty in the games to ensure a high level of challenges and motivation [52], while others prefer to perform different types of activities, as quoted: ‘*Doing my dusting and my polishing and things like that, is giving me more satisfaction than sitting down playing that [WiiTM]*.’ [50].

##### Usability

All qualitative studies showed that virtual reality exercises were easy to learn and flexible to use at any time of the day at home; however, (2/4) studies reported some technical issues that could affect the use of virtual reality games and exercises at home, as mentioned by one participant: “*it was very convenient. You could go over there in your robe or pajamas and do it if you didn’t want to get up at 8 o’clock in the morning …*” [49,50]. Setting up gloves before performing the exercises and changing batteries may take much time; the camera may occasionally fall during video conferencing, and sometimes there is no sufficient physical space at home [51,52]. Some patients experienced some technical problems related to the game itself in the form of false responses from the game, calibration of the avatar, screen freezing, and internet connection interruption [49,50].

##### Social Interaction

The qualitative results (1/4) reported that friends visiting or being away from home may hinder the ability to perform VR exercises [51]. Also, sometimes patients could stop using the device due to competing commitments, such as looking after their family members, or due to a limitation in their time due to returning to their pre-stroke lives [51].

##### Facilitators

In addition to patients’ experiences, there are a number of facilitators that could improve the adherence and participation of patients in their virtual reality telerehabilitation. These facilitators can be divided into two categories: internal motivation and external motivation. Internal motivation included some personal factors, as patients preferred to monitor their progress with exercises and improvements in their ADL and receive feedback from their therapist [52]. External motivation can be divided into three factors: training factors, social factors, and environmental factors. Training factors demonstrated that gamification of exercises can motivate patients to adhere to exercise programs by motivating them to win the games and obtain higher scores. Both the reactive and competitive nature of the games (tennis) could encourage patients to be more compliant with the exercises and to beat their previous score, as quoted: “*Yeah, well, I was trying to do that, beat the score from previous*.” [49,50,51]. In addition, the variation of games with different levels of difficulty could be a factor in motivating the patient to adhere more to exercises over time [52]. Social factors, including clinician encouragement via videoconferencing and motivational interviews and family and caregiver support, can motivate patients to engage more in performing their exercises at home, as quoted: “*My granddaughter used to play the Balloonpop and encouraged me. I mean, obviously she got fantastic scores that I wouldn’t be able to achieve, but I was so there, wanting to get as much as I could…*” [49,50]. Environmental factors that can influence patient motivation are location and time convenience, which lead to higher doses of exercise; some patients also prefer to perform exercises privately at home [50,51].

##### Barriers

There are some barriers that hinder the patients from participating in and adhering to the virtual reality exercises at home, which could be divided into four categories: personal, technical, environmental, and health barriers. Personal barriers: patients have mentioned that lack of motivation is the main cause of not adhering to exercises that have limited choices of exercises [49], limited difficulty levels and parameters of the games [49], and too easy games that they consider boring and less challenging to them [50]. Patients also reported that some exercises are generic and not tailored to their condition [50]. In addition, patients considered the gamified exercises to be childish games and preferred to perform more functional exercises [50]. Dependence on someone to help with equipment and unfamiliarity with technology can also affect the motivation of patients to perform their exercises, as quoted: “*If she (my daughter) wasn’t here, if she was at work, I’d used it later in the day when she came home.*” [49,51]. Technical barriers: patients reported that sometimes they spend too much time setting up the device, which could lead to performing fewer sessions [51,52]. Moreover, some patients reported issues related to disturbances in the internet connection [49]. One study reported that the use of the glove could be disturbed by bright sunlight or excessive infrared emission from other equipment in patients’ homes [51]. Health barriers: some patients were cautious about the risk of high blood pressure during exercises, which were considered more important for the patient than performing the exercises [50]. In addition, there were some health problems associated with performing exercises, like fatigue and depression, as quoted: “*In my first 4 months, I was really a bit tired every day. … I don’t think I’d have had the chance to do that (use the glove).*” [51]. Communication problems (aphasia) could affect patients’ abilities to perform videoconferencing with their clinician [49]. Environmental barriers: the limitation of physical space at patient homes [52].

## 4. Discussion

The purpose of this systematic review was to present the extent of evidence concerning the effectiveness of extended reality telerehabilitation and patients’ experiences of using different types of extended reality exercises at home. In our review, only one study had a sample size large enough to detect a small effect; however, there are some promising signals, with seven out of nine quantitative studies presenting an effect regarding the use of virtual reality and augmented reality exercises at home similar to in-clinic or other types of home exercises without any clear adverse effects and without any safety monitoring for the patients.

People after stroke raised concerns but also highlighted the potential of this rehabilitation technology; however, most notably, the current literature consisted mainly of low-quality, underpowered RCTs, which indicated a need to undertake higher-quality research with better monitoring of the intervention’s fidelity. This review presents more studies investigating the use of XR exercises even early in rehabilitation, with potential use for upper limb rehabilitation more than lower limb and balance rehabilitation.

We found that XR exercises were effective in the early subacute phase of stroke as well as the chronic stage. Han Young Jung (2017) reported that starting rehabilitation early after discharge is crucial in the recovery process after a stroke and can lead to better results and prognosis [53]. XR exercises can be effective with better recovery if implemented early in the subacute phase of home rehabilitation due to the spontaneous recovery of all functional outcomes within the early stage after stroke based on neural plasticity [54]. In the chronic stage (≥6 months post-stroke), although the effect of brain plasticity plateaus, VR home training can provide additional neural plasticity associated with functional improvement [54,55].

With regards to the power of this study, only one study was powered with a sample size of 230 to determine the effect; although the sample size was large, there was no significant difference between both interventions, and the effect size was trivial [44]. This may have been due to the use of non-specific VR games. In addition, there were two studies that reported large effects after using specific virtual reality exercises with a small sample size [36,47]. These findings could indicate a potential effect of these telerehabilitation interventions if they were included in a high-powered RCT. Our finding is in agreement with Maier et al. (2019), who found specific VR exercises were more effective than non-specific VR exercises compared to conventional therapy at a clinic. This is due to the greater effect of specific, tailored exercises based on neurological principles: task specificity, patient feedback, progression of exercises, functional tasks, and mechanisms of promoting the use of the affected limb [56].

Seven out of nine quantitative studies found that different types of XR exercises may be effective for different outcomes, with no data available regarding the quality of the evidence. Only one study reported no significant difference after using semi-immersive virtual reality exercises in hand rehabilitation [45]. Despite the majority of studies reporting an improvement in upper limb function with a non-significant difference compared to conventional therapy, there was a small effect size among all upper limb outcome measures. Our results agree with a recently updated Cochrane review that showed a low quality of evidence regarding the efficacy of telerehabilitation on different upper-limb functional outcomes compared to in-person rehabilitation or usual care for stroke patients [57]. Laver et al. (2020) included a variety of telerehabilitation interventions, including videoconferencing, videophones, web-based programs, virtual reality, and telemonitoring [57]. Furthermore, a narrative review with limited-strength evidence showed an improvement in upper-limb function after using VR-serious games as a tool of telerehabilitation; the motivational environment kept patients adherent to their exercises at home [58]. Uncertainty about the effectiveness of VR may be due to the small sample sizes of less-powered RCTs, which are the gold standard for clinical trials.

We found that only three out of nine quantitative studies included VR and AR balance exercises at home; two studies found that there was no significant difference compared to traditional balance exercises [36,46], and one study found that augmented reality was not effective at home among balance and gait outcomes [35]. Findings from a Cochrane review reported that there was weak evidence for the effect of any balance exercises in stroke telerehabilitation [57]. It is possible that these findings are due to the absence of a formal program of balance exercises with or without VR with high levels of difficulty that might be unsafe for stroke patients to perform alone at home. Moreover, performing balance exercises in the clinic with physiotherapist supervision improves the confidence of patients to perform more challenging and difficult levels of the exercises. On the other hand, another systematic review found that balance exercises in virtual reality combined with telerehabilitation balance training can be feasible for managing stroke patients at home with an improvement in balance outcome compared to balance training provided in the clinic by the therapists [59]. Schroder et al. (2019) included RCTs, pilot RCTs, case studies, and case-control studies in their review, which could influence the quality of the findings and results [59]. In addition, the main focus of their review was on feasibility rather than effectiveness.

Furthermore, another systematic review reported that virtual reality-based telerehabilitation had a significant effect on outcomes in upper limb function and balance in comparison to in-person rehabilitation at a clinic [60]. This review included studies with different locations for applying the intervention (hospital rooms simulating home environments, community sites, care facilities, and homes), which justify the difference between our results as patients could feel safer when performing the exercises at hospital or community sites as they will be more supported and supervised. In addition, this review did not provide any data regarding the monitoring of the rehabilitation program, adherence, or compliance of the patients.

Our review reported only four qualitative studies that examined patients’ opinions regarding rehabilitation of upper limbs and hands [49,50,51,52]. This limitation of the available data can influence the development, implementation, and dissemination of this technology. We found a variation in patients’ responses toward performing the virtual reality exercises at home. This variation may be due to the differences in behaviors and personalities among stroke patients that might be motivated by many gamification frameworks [61]. Most stroke gaming exercises focus on goal-oriented tasks, which could be suitable only for achiever types of people; however, variation in motivational factors should be considered for more adherence among different behavioral types of stroke patients [57].

Further, within our review, no studies had integrated progression. Evidence supports the need for repetitive practice to gain motor improvements, with 400 repetitions being a suggested amount [62]. Using different VR environments for training can influence physical improvement among stroke survivors. This may be due to the motivational and enjoyable environment that can encourage patient adherence [61]. The principles of neuroplasticity theory and motor learning and control theory support this improvement, as by increasing the number of repetitions of exercises, their brains can create new pathways and connections to help rebuild connections and improve overall functional outputs. In addition, these theories propose that repetition helps improve learning and the transfer of learned skills to other tasks [57,59]. In addition, using varieties of cueing (auditory and visual, direct from the therapist) during the execution of the exercises can help to enhance learning, skill acquisition, and functional outcomes based on motor learning theory [63,64,65]. No study utilized tailored gamified exercises based on the principles of neurological rehabilitation; most of the included gamified exercises used a popular platform that is not tailored specifically to stroke patients. Developing gamified exercise platforms that suit different stroke patients’ behaviors and interests can help stroke survivors perform more repetitions of exercises with high levels of satisfaction and motivation.

Based on these findings, it is recommended to use a user- or person-centered approach throughout the entire development process of such a complex XR intervention, considering all user opinions (patients, caregivers, and physiotherapists). It is therefore possible that the quality of the intervention can be influenced, resulting in better outcomes. Further studies could explore the reporting of intervention completion and fidelity and how to integrate this within the technology.

Regarding patients’ feedback on XR technology, none of the included studies used a framework to assess technology implementation, usability, and acceptability among stroke survivors that could influence the development and generalization of new versions of XR platforms. While implementation frameworks may have informed the design of the research that led to these studies, feasibility studies should consider implementation [66].

Our review and synthesis of qualitative studies reports that there may be a relationship between the complexity of the developed technology and the patient’s usability due to a lack of familiarity with technology, especially for elderly patients. This finding could stimulate the importance of including users’ opinions throughout the process of complex intervention development, taking into consideration the principles of implementing virtual care and technology in rehabilitation [61]. Currently, there is insufficient information regarding the long-term effects of home-based XR exercises. This lack of information may lead to a gap in our understanding of how these exercises affect different physical outcomes.

Based on our synthesis of the findings, XR-home-based exercises may enhance the rehabilitation program at home by providing a more convenient solution for people who are unable to access rehabilitation centers due to distance or time constraints. Additionally, it encourages stroke patients to practice a greater number of repetitions, thereby enabling physiotherapists to manage a large caseload of patients. This approach is in line with the WHO 2030 strategy to increase rehabilitation and utilize technology to address the global shortage of rehabilitation professionals. Our findings are in agreement with recently published reviews that found telerehabilitation using different tools can be effective for stroke patients’ rehabilitation [33,34,60]. On the other hand, some reviews reported weak evidence for using TR in stroke management [57,58].

Further research is needed to explore whether virtual reality and augmented reality exercise-based telerehabilitation affect different motor and non-motor outcomes and what patients’ and physiotherapists’ experiences and challenges are following the use of various XR platforms.

### Limitations

There are some limitations that should be highlighted in this review. Due to the inclusion of different types of XR exercises and the lack of standard outcome measures, it was not applicable to conduct a meta-analysis among different outcomes. In addition, due to the moderate to low quality of the included qualitative studies, it was not preferable to synthesize the qualitative outcomes using thematic synthesis. As the focus of this review was only on motor outcomes among the quantitative studies, less attention was given to non-motor outcomes (quality of life and cognition) and cost-effectiveness analyses that may influence the perception of exercise among patients. Due to differences between outcomes and outcome measures, we were unable to compare different types of XR platforms. Among the stroke survivors included in the eligible studies, none had severe cognitive or communication deficits, which may limit the generalization of our results to stroke survivors with good cognitive conditions and no communication disorders, which may not be the usual clinical presentation of stroke.

The qualitative studies included in our review focused primarily on patient experience, with less attention given to the experiences of physiotherapists and caregivers. Moreover, we did not compare the results across the different cultures regarding differences in the acceptance of technology use. All qualitative results are based on experiences with virtual reality exercises for upper limb management only, which might limit the extent to which they can be generalized. Another limitation of the review is that we only reported data within papers, and as such, any missing or unclear data is not reported, which may have affected results. Due to the variability in the reported outcomes, we decided to perform a narrative synthesis and were unable to perform any further analysis (meta-analysis). Moreover, some studies in our review did not provide comprehensive data, making a reliable certainty assessment challenging. Additionally, our review included various study designs that inherently present challenges in applying uniform certainty assessment methods, as they have different strengths and limitations.

## 5. Conclusions

Given the available evidence, this review provides a good understanding of the potential and promising effects of various types of extended reality and makes recommendations for its integration into rehabilitation as a feasible and potential tool for stroke rehabilitation at home and in community settings. To date, most studies are on the upper limb, and there is only one RCT with a sample greater than 200, highlighting the need to establish safety and feasibility for a wider range of rehabilitation and for future fully powered trials. Further, we observed limited reporting of intervention adherence and compliance to dosage, and no studies integrated movement quality and monitored patients’ safety during the execution of the exercises alone at home.

## Figures and Tables

**Table 1:** Risk of bias in the qualitative studies.

CASP Critical Appraisal Tool	Allegue, 2022 [49]	Wingham, 2015 [50]	Standen, 2014 [51]	Chen, 2020 [52]
Was there a clear statement of the aims of the research?	Yes	Yes	Yes	Yes
Is a qualitative methodology appropriate?	Yes	Yes	Yes	Yes
Was the research design appropriate to address the aims of the research?	Yes	Yes	Cannot tell	Cannot tell
Was the recruitment strategy appropriate to the aims of the research?	Yes	Yes	Cannot tell	Yes
Was the data collected in a way that addressed the research issue?	Yes	Cannot tell	Cannot tell	Cannot tell
Has the relationship between researcher and participants been adequately considered?	Cannot tell	Cannot tell	Cannot tell	Cannot tell
Have ethical issues been taken into consideration?	Cannot tell	Cannot tell	Cannot tell	no
Was the data analysis sufficiently rigorous?	Yes	Yes	No	Yes
Is there a clear statement of findings?	Cannot tell	Cannot tell	No	Yes
How valuable is the research?	Yes	yes	Cannot tell	yes
Score: Yes: 1 point; Cannot tell: 0.5 point; No: 0 point. If 9–10 (high quality) If 7.5–9 (moderate quality) If less than 7.5 (low quality)	8.5 (moderate)	8 (moderate)	5 (low quality)	7.5 (moderate)

## Data Availability

Data are contained within this article or the Supplementary Materials.

## Supplementary Materials

The following supporting information can be downloaded at: https://www.mdpi.com/article/10.3390/ijerph20176630/s1. Table S1: search keywords, Table S2: search strategy; Table S3: summary of effect size, Table S4: Virtual reality exercises used in the experimental groups, Table S5: Summary of the quantitative studies, Table S6: Summary of quantitative outcomes, Table S7: summary of the quantitative outcome effect measures, Table S8: Summary of qualitative studies characteristics [67,68,69,70,71,72,73,74].
