# Supplementary material for: The Extent of Evidence Supporting the Effectiveness of Extended Reality Telerehabilitation on Different Qualitative and Quantitative Outcomes in Stroke Survivors: A Systematic Review
Source: Int J Environ Res Public Health. 2023 Aug 23;20(17):6630. doi: 10.3390/ijerph20176630 (PMC10487831; doi:10.3390/ijerph20176630)
Supplement: Supplementary file 1 [file ijerph-20-06630-s001.zip › supplementary documents (final).pdf]

Table S1 :Search keywords

Keywords	Alternatives
Population (stroke)	“Stroke” OR cerebrovascular accident* OR brain vascular accident* OR “cerebrovascular stroke” OR “cerebral stroke” OR “acute stroke” OR “chronic stroke” OR “hemiplegic “OR “hemiparesis” OR “paresis” OR ‘’CVA’’ OR ‘’CVAs’’ OR “transient ischemic attack*” OR “haemorrhagic stroke “ OR “Subarachnoid Haemorrhagic Stroke” OR “subarachnoid stroke” OR “Intracerebral Hemorrhagic Stroke” OR “hemorrhagic stroke” OR “subarachnoid hemorrhagic stroke” OR traumatic brain injur*
Intervention (virtual reality exercises)	“virtual reality exercise” OR "Augmented Reality" OR "Virtual Reality" OR “virtual environment” OR “VR” OR “AR” OR “VR-based treadmill training “ OR exergame* OR “wii” OR “Kinect” OR “Immersive VR” OR “Immersion VR” OR “Non-immersive VR” OR “Non-immersion VR” OR “Non-immersive virtual reality” OR “gamification” OR game* OR “wii” OR “mixed – augmented” OR augmented realit* OR mixed realit* OR extended realit* OR "360 video" OR "computer simulation" OR video game* OR “virtual environment” OR “serious game” OR “immersive environment” OR "Xbox Kinect" OR “Nintendo” OR “Active-Video Gaming” OR Virtual Reality Exercise*
Outcomes<br>Motor and qualitative outcomes	"motor" OR “mobility" OR “function” OR “equilibrium” OR "balance " OR “posture” OR "gait" OR “gait analysis” OR “walking” OR “speed” OR “stability” OR “posture control” OR “upper limb function*” OR “arm” OR “hand” OR “dexterity” OR lower limb function* OR “trunk” OR “trunk control” OR “core” OR “proprioception” OR “coordination” OR “physical” OR “ADL” OR “daily living activit*” OR “activit* of daily living” OR “motivation” OR “quality of life” OR “QoL” OR “performance” OR “cost effect*” OR “cost”<br>Experience* OR “feedback” OR view* OR “opinions” OR “needs” OR facilitator* OR barrier* OR expectation* OR “compliance” OR “non-compliance” OR concern* OR satisfaction* OR dissatisfaction* OR accept* OR “safety” OR “perception” OR perceive* OR “performance” OR “motivation” OR performance* OR “complexity” OR “qualitative research” OR “qualitative method*” OR interview* OR "focus group*" OR “qualitative”
Setting<br>telerehabilitation	“Home rehabilitation” OR “home therapy” OR “telerehabilitation” OR<br>“Tele-rehabilitation” OR

	“Remote Rehabilitation”<br>“Virtual Rehabilitation” OR<br>“home” OR resident OR “house”

Outcomes	Operational Definition
Upper limb function	The ability to move the upper limb and do all functional activities in good quality and range of motion including both gross and fine motor skills [67].
Lower limb function	The ability to move the lower limb and do all functional activities (sit to stand , standing , walking) in good quality and range of motion [68].
Balance	The ability to maintain centre of gravity within the base of support during both static and dynamic positions [69].
Muscle strength	The amount of force required by a single or a group of muscles to do a certain activity [70].
Mobility	The ability of the person to move and transfer freely to perform different functional tasks [71].
Risk of fall	The probability of a person to fall which may be due to different causes (motor, neurological, cognitive, visual , environmental , psychological) [72].
Gait	The bipedal locomotion function of the lower limb including pattern of coordinated movements of the different body parts [73].
Experiences	The patients’ perception toward certain types of activities or illness and considered to be a component of health care service[74].
Facilitators	Factors that encourage the people from doing certain activities[74].
Barriers	Factors that hinder the people from doing certain activities[74].

Table S2: Search strategy

Database	Strategy	Number 1/2023
Pubmed	(("Stroke" OR cerebrovascular accident* OR brain vascular accident* OR "cerebrovascular stroke" OR "cerebral stroke" OR "acute stroke" OR "chronic stroke" OR "hemiplegic "OR "hemiparesis" OR "paresis" OR ‘’CVA’’ OR ‘’CVAs’’ OR "transient ischemic attack*" OR "haemorrhagic stroke " OR "Subarachnoid Haemorrhagic Stroke" OR "subarachnoid stroke" OR "Intracerebral Hemorrhagic Stroke" OR "hemorrhagic stroke" OR "subarachnoid hemorrhagic stroke" OR traumatic brain injur*) AND ("virtual reality exercise" OR "Augmented Reality" OR "Virtual Reality" OR "virtual environment" OR "VR" OR "AR" OR "VR-based treadmill training " OR exergame* OR "wii" OR "Kinect" OR "Immersive VR" OR "Immersion VR" OR "Non-immersive VR" OR "Non-immersion	295

	VR" OR "Non-immersive virtual reality" OR "gamification" OR game* OR "wii" OR "mixed – augmented" OR augmented realit* OR mixed realit* OR extended realit* OR "360 video" OR "computer simulation" OR video game* OR "virtual environment" OR "serious game" OR "immersive environment" OR "Xbox Kinect" OR "Nintendo" OR "Active-Video Gaming" OR Virtual Reality Exercise*)) AND ("motor" OR "mobility" OR "function" OR "equilibrium" OR "balance " OR "posture" OR "gait" OR "gait analysis" OR "walking" OR "speed" OR "stability" OR "posture control" OR "upper limb function*" OR "arm" OR "hand" OR "dexterity" OR lower limb function* OR "trunk" OR "trunk control" OR "core" OR "proprioception" OR "coordination" OR "physical" OR "ADL" OR "daily living activit*" OR "activit* of daily living" OR "motivation" OR "quality of life" OR "QoL" OR "performance" OR "cost effect*" OR "cost" Experience* OR "feedback" OR view* OR "opinions" OR "needs" OR facilitator* OR barrier* OR expectation* OR "compliance" OR "non-compliance" OR concern* OR satisfaction* OR dissatisfaction* OR accept* OR "safety" OR "perception" OR perceive* OR "performance" OR "motivation" OR performance* OR "complexity" OR "qualitative research" OR "qualitative method*" OR interview* OR "focus group*" OR "qualitative")) AND ("Home rehabilitation" OR "home therapy" OR "telerehabilitation" OR "Tele-rehabilitation" OR "Remote Rehabilitation" "Virtual Rehabilitation" OR "home" OR resident OR "house")	
WoS	(((TS=("Stroke" OR cerebrovascular acciden* OR brain vascular acciden* OR "cerebrovascular stroke" OR "cerebral stroke" OR "acute stroke" OR "chronic stroke" OR "hemiplegic "OR "hemiparesis" OR "paresis" OR ''CVA'' OR ''CVAs'' OR "transient ischemic attack" OR "haemorrhagic stroke " OR "Subarachnoid Haemorrhagic Stroke" OR "subarachnoid stroke" OR "Intracerebral Hemorrhagic Stroke" OR "hemorrhagic stroke" OR "subarachnoid hemorrhagic stroke" OR traumatic brain injur*)) AND TS=("virtual reality exercise" OR "Augmented Reality" OR "Virtual Reality" OR "virtual environment" OR "VR" OR "AR" OR "VR-based treadmill training " OR exergame* OR "wii" OR "Kinect" OR "Immersive VR" OR "Immersion VR" OR "Non-immersive VR" OR "Non-immersion VR" OR "Non-immersive virtual reality" OR "gamification" OR game* OR "wii" OR "mixed – augmented" OR virtual realit* OR augmented realit* OR mixed realit* OR extended realit* OR "360 video" OR "computer simulation" OR video game* OR "virtual environment" OR "serious game" OR "immersive environment" OR "Xbox Kinect" OR "Nintendo" OR "Active-Video Gaming" OR Virtual Reality Exercis*)) AND TS=("motor" OR "mobility" OR "function" OR "equilibrium" OR "balance " OR "posture" OR "gait" OR "gait analysis" OR "walking" OR "speed" OR "stability" OR "posture control" OR upper limb functio* OR "arm" OR "hand" OR "dexterity" OR lower limb functio* OR "trunk" OR "trunk control" OR "core" OR "proprioception" OR "coordination" OR "physical" OR "ADL" OR daily living activit* OR activit* of daily living OR "motivation" OR "quality of life" OR "QoL" OR "performance" OR cost effect* OR "cost" Experience* OR "feedback" OR view* OR "opinions"OR "needs" OR facilitato* OR barrie* OR expectatio* OR "compliance" OR "non-compliance" OR concern* OR satisfaction* OR dissatisfaction* OR accept* OR concern* OR "safety" OR "perception" OR perceive* OR "performance" OR "motivation" OR performance* OR "complexity" OR "qualitative research" OR "qualitative method*" OR interview* OR "focus group*" OR "qualitative")) AND TS=("Home rehabilitation" OR "home therapy" OR "telerehabilitation" OR "Tele-rehabilitation" OR "Remote Rehabilitation" "Virtual Rehabilitation" OR "home" OR "resident" OR "house")	967

CINAHL	(“Stroke” OR cerebrovascular accident* OR brain vascular accident* OR “cerebrovascular stroke” OR “cerebral stroke” OR “acute stroke” OR “chronic stroke” OR “hemiplegic “OR “hemiparesis” OR “paresis” OR ‘’CVA’’ OR ‘’CVAs’’ OR “transient ischemic attack*” OR “haemorrhagic stroke “ OR “Subarachnoid Haemorrhagic Stroke” OR “subarachnoid stroke” OR “Intracerebral Hemorrhagic Stroke” OR “hemorrhagic stroke” OR “subarachnoid hemorrhagic stroke” OR traumatic brain injur*) AND (“virtual reality exercise” OR "Augmented Reality" OR "Virtual Reality" OR “virtual environment” OR “VR” OR “AR” OR “VR-based treadmill training “ OR exergame* OR “wii” OR “Kinect” OR “Immersive VR” OR “Immersion VR” OR “Non-immersive VR” OR “Non-immersion VR” OR “Non-immersive virtual reality” OR “gamification” OR game* OR “wii” OR “mixed – augmented” OR augmented realit* OR mixed realit* OR extended realit* OR "360 video" OR "computer simulation" OR video game* OR “virtual environment” OR “serious game” OR “immersive environment” OR "Xbox Kinect" OR “Nintendo” OR “Active-Video Gaming” OR Virtual Reality Exercise*) AND ("motor" OR “mobility" OR “function” OR “equilibrium” OR "balance " OR “posture” OR "gait" OR “gait analysis” OR “walking” OR “speed” OR “stability” OR “posture control” OR “upper limb function*” OR “arm” OR “hand” OR “dexterity” OR lower limb function* OR “trunk” OR “trunk control” OR “core” OR “proprioception” OR “coordination” OR “physical” OR “ADL” OR “daily living activit*” OR “activit* of daily living” OR “motivation” OR “quality of life” OR “QoL” OR “performance” OR “cost effect*” OR “cost” Experience* OR “feedback” OR view* OR “opinions” OR “needs” OR facilitator* OR barrier* OR expectation* OR “compliance” OR “non-compliance” OR concern* OR satisfaction* OR dissatisfaction* OR accept* OR “safety” OR “perception” OR perceive* OR “performance” OR “motivation” OR performance* OR “complexity” OR “qualitative research” OR “qualitative method*” OR interview* OR "focus group*" OR “qualitative”) AND (“Home rehabilitation” OR “home therapy” OR “telerehabilitation” OR “Tele-rehabilitation” OR “Remote Rehabilitation” “Virtual Rehabilitation” OR “home” OR resident OR “house”)<br>**Limiters - Language**: English<br>**Expanders -** Apply equivalent subjects<br>**Search modes** - Boolean/Phrase	174
Medline	Ovid MEDLINE(R) ALL <1946 to January 06, 2023><br>1 stroke.mp. [mp=title, book title, abstract, original title, name of substance word, subject heading word, floating sub-heading word, keyword heading word, organism supplementary concept word, protocol supplementary concept word, rare disease supplementary concept word, unique identifier, synonyms] 357938<br>2 cerebrovascular accident.mp. [mp=title, book title, abstract, original title, name of substance word, subject heading word, floating sub-heading word, keyword heading word, organism supplementary concept word, protocol supplementary concept word, rare disease supplementary concept word, unique identifier, synonyms] 5327	369

	3 Stroke/ or Hemiplegia/ 137643<br>4 Exergaming/ 92<br>5 Virtual Reality/ or Stroke Rehabilitation/ 21703<br>6 virtual reality exercises.mp. [mp=title, book title, abstract, original title, name of substance word, subject heading word, floating sub-heading word, keyword heading word, organism supplementary concept word, protocol supplementary concept word, rare disease supplementary concept word, unique identifier, synonyms] 31<br>7 Augmented Reality/ 1020<br>8 augmented reality.mp. [mp=title, book title, abstract, original title, name of substance word, subject heading word, floating sub-heading word, keyword heading word, organism supplementary concept word, protocol supplementary concept word, rare disease supplementary concept word, unique identifier, synonyms] 4018<br>9 immersive virtual reality.mp. 1064<br>10 non-immersive virtual reality.mp. 72<br>11 1 or 2 or 3 370289<br>12 4 or 5 or 6 or 7 or 8 or 9 or 10 26011<br>13 Motor Activity/ or motor outcomes.mp. 100740<br>14 "Activities of Daily Living"/ or mobility function.mp. 72080<br>15 balance.mp. or Postural Balance/ 302226<br>16 Gait/ or Gait Analysis/ or gait.mp. 73717<br>17 Upper Extremity/ or upper limb function.mp. 15471<br>18 walking.mp. or Walking Speed/ or Walking/ 99997<br>19 Hand Strength/ or Motor Skills/ or hand dexterity.mp. 45003<br>20 trunk control.mp. 673<br>21 proprioception.mp. or Proprioception/ 12141	

	22 Accidental Falls/ or risk of fall.mp. 28179<br>23 quality of life.mp. or "Quality of Life"/ 431548<br>24 Qualitative Research/ 78604<br>25 Patient Satisfaction/ or patients views.mp. 89985<br>26 experiences.mp. 258545<br>27 facilitators.mp. 23823<br>28 expectations.mp. or Motivation/152307<br>29 barriers.mp. 187125<br>30 compliance.mp. or Patient Compliance/ 185866<br>31 cost effectiveness.mp. or Cost-Benefit Analysis/ 126370<br>32 13 or 14 or 15 or 16 or 17 or 18 or 19 or 20 or 21 or 22 or 23 or 24 or 25 or 26 or 27 or 28 or 29 or 30 or 31 1941267<br>33 telerehabilitation.mp. or Telerehabilitation/ or Rehabilitation/ or Remote Consultation/ 26158<br>34 home rehabilitation.mp. 524<br>35 33 or 34 26594<br>36 11 and 12 and 32 and 35 369	
Embase	Embase <1974 to 2023 January 06><br><br>1 stroke.mp. [mp=title, abstract, heading word, drug trade name, original title, device manufacturer, drug manufacturer, device trade name, keyword heading word, floating subheading word, candidate term word] 536823<br>2 cerebrovascular accident.mp. [mp=title, abstract, heading word, drug trade name, original title, device manufacturer, drug manufacturer, device trade name, keyword heading word, floating subheading word, candidate term word] 270659	858

	3 Stroke/ or Hemiplegia/ 198051<br>4 Exergaming/ 194<br>5 Virtual Reality/ or Stroke Rehabilitation/ 30547<br>6 virtual reality exercises.mp. [mp=title, abstract, heading word, drug trade name, original title, device manufacturer, drug manufacturer, device trade name, keyword heading word, floating subheading word, candidate term word] 44<br>7 Augmented Reality/ 1772<br>8 augmented reality.mp. [mp=title, abstract, heading word, drug trade name, original title, device manufacturer, drug manufacturer, device trade name, keyword heading word, floating subheading word, candidate term word] 4768<br>9 immersive virtual reality.mp. 1192<br>10 non-immersive virtual reality.mp. 88<br>11 1 or 2 or 3 622687<br>12 4 or 5 or 6 or 7 or 8 or 9 or 10 34580<br>13 Motor Activity/ or motor outcomes.mp. 48271<br>14 "Activities of Daily Living"/ or mobility function.mp. 88101<br>15 balance.mp. or Postural Balance/ 391093<br>16 Gait/ or Gait Analysis/ or gait.mp. 119268<br>17 Upper Extremity/ or upper limb function.mp. 23680<br>18 walking.mp. or Walking Speed/ or Walking/ 164935<br>19 Hand Strength/ or Motor Skills/ or hand dexterity.mp. 88393<br>20 trunk control.mp. 970<br>21 proprioception.mp. or Proprioception/ 17798	

	22 Accidental Falls/ or risk of fall.mp. 44250<br>23 quality of life.mp. or "Quality of Life"/ 751457<br>24 Qualitative Research/ 108748<br>25 Patient Satisfaction/ or patients views.mp. 164604<br>26 experiences.mp. 304397<br>27 facilitators.mp. 30117<br>28 expectations.mp. or Motivation/213956<br>29 barriers.mp. 229375<br>30 compliance.mp. or Patient Compliance/ 386445<br>31 cost effectiveness.mp. or Cost-Benefit Analysis/ 277085<br>32 13 or 14 or 15 or 16 or 17 or 18 or 19 or 20 or 21 or 22 or 23 or 24 or 25 or 26 or 27 or 28 or 29 or 30 or 31 2897880<br>33 telerehabilitation.mp. or Telerehabilitation/ or Rehabilitation/ or Remote Consultation/ 108458<br>34 home rehabilitation.mp. 1445<br>35 33 or 34 109575<br>36 11 and 12 and 32 and 35 858	
PEDro	Stroke AND telerehabilitation<br>**Excluding** systematic reviews.	20
Registered trials	“Stroke” OR cerebrovascular acciden* OR brain vascular acciden* OR “cerebrovascular stroke” OR “cerebral stroke” OR “acute stroke” OR “chronic stroke” OR “hemiplegic “OR “hemiparesis” OR “paresis” OR ‘’CVA’’ OR ‘’CVAs’’ OR “transient ischemic attac | stroke | “virtual reality exercise” OR "Augmented Reality" OR "Virtual Reality" OR “virtual environment” OR “VR” OR “AR” OR “VR-based treadmill training “ OR exergame* OR “wii” OR “Kinect” OR “Immersive VR” OR “Immersion VR” OR “Non-immersive VR” OR “Non-imme | “upper limb function” OR “lower limb	5

	function" OR "balance" OR "ADL" OR "mobility" OR "truck control" OR "gait" OR "falls" OR "quality of life" OR "experiences" OR "needs" OR "expectations" OR "facilitators" OR "barriers" OR "qualitative studie* | Adult, Older Adult<br>**Applied Filters**: Adult (18–64) Older Adult (65+) , has results.	
ProQuest Dissertations & Theses Global	summary("Stroke" OR cerebrovascular acciden* OR brain vascular acciden* OR "cerebrovascular stroke" OR "cerebral stroke" OR "acute stroke" OR "chronic stroke" OR "hemiplegic " OR "hemiparesis" OR "paresis" OR ''CVA'' OR ''CVAs'' OR "transient ischemic attack" OR "haemorrhagic stroke " OR "Subarachnoid Haemorrhagic Stroke" OR "subarachnoid stroke" OR "Intracerebral Hemorrhagic Stroke" OR "hemorrhagic stroke" OR "subarachnoid hemorrhagic stroke" OR traumatic brain injur*) AND summary("virtual reality exercise" OR "Augmented Reality" OR "Virtual Reality" OR "virtual environment" OR "VR" OR "AR" OR "VR-based treadmill training " OR exergame* OR "wii" OR "Kinect" OR "Immersive VR" OR "Immersion VR" OR "Non-immersive VR" OR "Non-immersion VR" OR "Non-immersive virtual reality" OR "gamification" OR game* OR "wii" OR "mixed augmented" OR virtual realit* OR augmented realit* OR mixed realit* OR extended realit* OR "360 video" OR "computer simulation" OR video game* OR "virtual environment" OR "serious game" OR "immersive environment" OR "Xbox Kinect" OR "Nintendo" OR "Active-Video Gaming" OR Virtual Reality Exercis*) AND summary("motor" OR "mobility" OR "function" OR "equilibrium" OR "balance " OR "posture" OR "gait" OR "gait analysis" OR "walking" OR "speed" OR "stability" OR "posture control" OR "upper limb functio*" OR "arm" OR "hand" OR "dexterity" OR lower limb functio* OR "trunk" OR "trunk control" OR "core" OR "proprioception" OR "coordination" OR "physical" OR "ADL" OR "daily living activit*" OR "activit* of daily living" OR "motivation" OR "quality of life" OR "QoL" OR "performance" OR "cost effect*" OR "cost" Experience* OR "feedback" OR view* OR "opinions" OR "needs" OR facilitato* OR barrie* OR expectatio* OR "compliance" OR "non-compliance" OR concern* OR satisfaction* OR dissatisfaction* OR accept* OR "safety" OR "perception" OR perceive* OR "performance" OR "motivation" OR performance* OR "complexity" OR "qualitative research" OR "qualitative method*" OR interview* OR "focus group*" OR "qualitative") AND summary("Home rehabilitation" OR "home therapy" OR "telerehabilitation" OR "Tele-rehabilitation" OR "Remote Rehabilitation" "Virtual Rehabilitation" OR "home" OR "resident" OR "house")<br>**limited by English language**	49

Table S3: Summary of effect sizes .

				Outcome measures																	
Study		Sample size		Upper limb function								Lower limb function	Muscle strength		Mobility		Balance		Gait		Fall
			FOLLOW UP	FMA-UE	ARAT	BBT	Stroke Impact Scale hand motor domain scores	Nine Hole Peg Test	CAHAI	MAL (quality of movement)	MAL (amount of use)	FMA-LE	MRC proximal	MRC distal	TUG:	POMA	POMA-B	BBS	POMA-G	FAC	FES-I
Cramer ., 2019		124	baseline to 30 days	-1.1			X														
Yang ., 2022		84	baseline-post	-0.49								0.05						0.16		0.24*	
Adie ., 2017		235	baseline-post (6 weeks)		-0.75																
			baseline-post (6 months)		-0.68																
Ballester ., 2017		35	baseline-post (week 3)	−0.30					0.48*				−0.17	−0.12							
			baseline-post (week 12)	0.34*					−0.08				−0.62	−0.17							
Lee ., 2022		68	baseline - post (4 weeks)												X	X		X			X
			baseline - follow up (4 weeks)												X	X		X			X
Piron ., 2009		36	T30-T60	0.30*																	

			T60-T90	0.32*																	
Llore´ns ., 2015		30	base line to week 8														0.54**	0.66**	0.89***		
			week 8 to week 12														0.92***	-0.75	-0.15		
Zondervan ., 2016		17	baseline-1 month post treatment		0.25*	-0.35		0.23*		1.37****	0.90***										
Hernandez ., 2022		51	NO DATA AVAILABLE	X																	

All numbers represent Cohen's d.(Effect size)

Classification of effect sizes: trivial (Cohen's d ≤ .2); * small (Cohen's d > .2); ** moderate (Cohen's d > .5); *** large (Cohen's d > .8); **** very large (Cohen's d > 1.3).

(X) means impossible to calculate effect size based on reported results.

Table S4: Virtual reality exercises used in the experimental groups.

Study	VR exercise	Mode of delivery
cramer.,2019	Arm exercises (88 exercises) using standard exercise equipment (resistance tubing or putty) including (stretching, strengthening, and active ROM).(SVR)	Non-immersive VR
Yang.,2022	Exercises in form of the upper limb (22 exercises) and lower limb (7 exercises) motor functions, balance ability (11 exercises), or/and coordination (6 exercises).(SVR)	AR
Adie., 2017	Wii sports games (bowling, tennis, golf, and baseball) (NSVR)	Non-immersive VR
Ballester ., 2017	goal-directed grasp action execution and motor imagery.(SVR)	Semi-immersive VR

Lee ., 2022	endurance, speed, and range of motion of the upper extremities, trunk, hips, and whole body.(SVR)	AR
Piron ., 2009	5 virtual motor tasks. (SVR)	Non-immersive VR
Llore´ns ., 2015 [15]	Balance exercises in empty scenario environment.(SVR)	Non-immersive VR
Zondervan ., 2016 [16]	MusicGlove therapy.(SVR)	Semi-immersive VR
Hernandez ., 2022 [17]	Jintronix system (NSVR)	Non-immersive VR

Table S5: Summary of the quantitative studies.

Study author & year (country)	design	Participants' number (age)	Time from a stroke in days(stage)	Stroke subtype in %	Cognitive state	Intervention	Setting of treatment	Revision of treatment plan	Dose of exercises	multimorbid conditions (event/total)	Target region	Devices used to deliver VR exercises	Primary Outcomes (Outcome measure scale)	Adverse effects
Cramer .,2019 (USA)	RCT	Total:124<br>E:62 (62±14)<br>C:62 (60±13)	E: 132 ±65<br>C: 129±59<br>Patients enrolled <90 d after stroke<br>E: 16 ±25.8.<br>C: 22 ±35.5<br>(Subacute – chronic)	E: ischemic 87.1%<br>Hemorrhagic 12.9%<br>C: ischemic 83.9%<br>hemorrhagic 16.1%	Excluded in there was a Significant cognitive impairment, defined as Montreal Cognitive Assessment score < 22 (a lower score was permitted if due to aphasia	EG: VR exercises<br>CG: arm exercises (demonstrated by the therapists on supervised days or via printed homework on unsupervised days).	EG: at home.<br>CG: 18 supervised sessions at research centre and 18 unsupervised sessions at home.	every 2 weeks	EG:<br>(6-8 weeks)<br>(36 sessions)<br>(70 minutes plus a 10-minute break; 18 supervised and 18 unsupervised) period. CC:<br>(6-8 weeks)<br>(36 sessions)	Hypertension<br>E: (50/62)<br>C: (53/62)<br>Diabetes mellitus<br>E: (14/62)<br>C: (17/62)<br>Atrial fibrillation<br>E: (10/62)<br>C: (4/62)<br>Hypercholesterolemia<br>E: (40/62)<br>C: (39/62)	UL	Table chair<br>Computer with monitor, microphone, and speakers<br>Verizon wireless modem<br>Myo Band<br>Wiimote in a pistol-shaped holder	Upper limb function (FMA-UE)	Non-serious adverse Events:<br>6 patients in the experimental group complained from arm and shoulder pain and 5 patients in the control group complained from fatigue and arm and shoulder pain.

Study author & year (country)	design	Participants' number (age)	Time from a stroke in days(stage)	Stroke subtype in %	Cognitive state	Intervention	Setting of treatment	Revision of treatment plan	Dose of exercises	multimorbid conditions (event/total)	Target region	Devices used to deliver VR exercises	Primary Outcomes (Outcome measure scale)	Adverse effects
					and if allowed by the site PI)				(70 minutes plus a 10-minute break; 18 supervised and 18 unsupervised)			Power Mate PlayStation 3 Eye Move Controller Joystick Logitech Trackpad Standard rehabilitation therapy devices for the upper extremity		
Yang.,2022 (Hong Kong)	RCT	Total ITT=84<br>PP=39<br>E:16 (64.61±9.9)<br>C:23 (62.79±13.79)	E: 1339.5±974.5<br>C: 1244.6±992.8<br>(chronic)	E: ischemic 68.75%<br>Hemorrhagic 31.25%<br>C: ischemic 65.21%<br>Hemorrhagic 34.78%	Patient must have sufficient cognition to follow the instructions provided by the therapists and the computer	EG: AR exercises in form of the upper-limb (22 exercises)<br>and lower-limb (7 exercises) motor functions, balance<br>ability (11 exercises),<br>or/and coordination (6 exercises) at home .	EG:at home / CG:at rehabilitation centre	weekly	EG:<br>(4-10 weeks)<br>20 sessions (2-5 per week)<br>120 min per session (2-10 exercises / session).	NR	UL, LL, Trunk	Microsoft Kinect, a TV/Monitor, and an AR rehabilitation software platform.	Upper limb function (FMA-UE)<br>Lower limb function (FMA-LE),<br>Gait	NR

Study author & year (country)	design	Participants' number (age)	Time from a stroke in days(stage)	Stroke subtype in %	Cognitive state	Intervention	Setting of treatment	Revision of treatment plan	Dose of exercises	multimorbid conditions (event/total)	Target region	Devices used to deliver VR exercises	Primary Outcomes (Outcome measure scale)	Adverse effects
						**supervised** While the participant was taking the training at home, the human trainer could monitor the training process remotely.<br>CG: AR exercises in form of the upper-limb (22 exercises)<br>and lower-limb (7 exercises) motor functions, balance<br>ability (11 exercises), or/and coordination (6 exercises) at the rehabilitation centres<br>, with 3/4 of each session delivered<br>by a human trainer and the rest of 1/4 delivered by a virtual trainer.			CG:(4-10 weeks)<br>20 sessions (2-5 per week)<br>120 min per session (2-10 exercises / session).				(FAC),<br>Balance (BBS)	

Study author & year (country)	design	Participants' number (age)	Time from a stroke in days(stage)	Stroke subtype in %	Cognitive state	Intervention	Setting of treatment	Revision of treatment plan	Dose of exercises	multimorbid conditions (event/total)	Target region	Devices used to deliver VR exercises	Primary Outcomes (Outcome measure scale)	Adverse effects
Adie., 2017 (UK)	RCT	Total=235<br>E:117 (66.8±14.6)<br>C:118 (68±11.9)	E:57.3±48.3<br>C:56.3±50.1<br>(chronic)	E: ischemic 88.88%<br>Hemorrhagic 11.12%<br>C: ischemic 88.9%<br>Hemorrhagic 11.1%	NR	EG: WiiTM sports games (bowling, tennis, golf and baseball in a seated position<br>CG: tailored arm exercises (based on the Graded Repetitive Arm Supplementary Programme) in a seated position , **(unsupervised exercises).**	EG:at home / CG: at home.	weekly	EG:<br>(6 weeks)<br>(Daily sessions)<br>(45 min / session) in a seated position.<br>CG:<br>(6 weeks)<br>(Daily sessions)<br>(45 min / session) in a seated position.	diabetes mellitus<br>E: (18/117)<br>C: (26/118)<br>cardiovascular<br>E: (43/117)<br>C: (57/118)<br>respiratory disease<br>E: (25/117)<br>C: (12/118)	UL	computer screen - remote - camera - Nintendo Wii Sports	Upper limb function (ARAT)	NR
Ballester ., 2017 (Spain)	RCT	Total=35<br>E:17 (65.05± 10.33)<br>C:18 (61.75± 12.94)	E:1073.43±767.74<br>C: 798.06 ±421.80<br>(chronic)	E: ischemic 67%<br>Haemorrhagic 33%<br>C: ischemic 67%<br>Haemorrhagic 33%	Mini-Mental State Evaluation, MMSE>22.	EG:The RGS integrates a paradigm of goal-directed action execution and motor imagery **(unsupervised exercises).**<br>CG:horizontal and vertical stacking and unstacking of plastic cups with their right and left hand consecutively	EG: Domiciliary<br>CG: Domiciliary	NM	EG:<br>(3 weeks)<br>weekly days,<br>(20 minutes / session). CG:<br>(3 weeks)<br>Weekly days,<br>(20 minutes /session).	NM	UL	pair of data gloves equipped with bend sensors, screen , camera	Upper limb function (FMA-UE) ,(CAHAI).	NR

Study author & year (country)	design	Participants' number (age)	Time from a stroke in days(stage)	Stroke subtype in %	Cognitive state	Intervention	Setting of treatment	Revision of treatment plan	Dose of exercises	multimorbid conditions (event/total)	Target region	Devices used to deliver VR exercises	Primary Outcomes (Outcome measure scale)	Adverse effects
						without assisstence.**(unsupervised exercises)**								
Lee ., 2022 (Republic of Korea)	RCT	ITT=56<br>E:29 (60.4±14.4)<br>C:27<br>(66.1±10.6)	E: 52.5 ±56.0<br>C: 33.4 ±51.6<br>(subacute , chronic)	E: ischemic 86.2%<br>Haemorrhagic 13.8%<br>C: ischemic 96.3%<br>Haemorrhagic 3.7%	Mini-Mental State Evaluation, MMSE>22.	EG:UINCARE HomeVR device at home , to train the control, endurance, speed, accuracy, and range of motion of the upper extremities and trunk , hips and whole body in form of 10 levels of progression.<br>CG:conventional exercise program (stretching, aerobic, and strengthening exercises with a written and pictorial HEP	EG:at home,<br>CG:at home	weekly	EG :<br>(4 weeks)<br>5 sessions / week. (30min / session)<br>CG:<br>(4 weeks)<br>5 sessions / week. (30min / session)	NR	Whole body	sensors , camera , computer screen	Mobility (TUG, POMA)<br>Balance (BBS) Fear of fall (FES-I)	1 participant complained from infratentorial ischemic stroke due to vertebral artery dissection (TIA) after performing excessive head rotation during a rotation exercise of the trunk for 3 weeks.
Piron ., 2009 (Italy)	RCT	Total=36<br>E:18 (66±7.9)<br>C:18 (64.4±7.9)	E:445.3±200.75<br>C:361.3±112.42<br>(chronic)	E: ischemic 100%<br>C: ischemic 100%	Patients with clinical evidence of cognitive impairment, such as apraxia (score	EG: 5 virtual motor tasks / supervised via videoconferencing.<br>CG:conventional physiotherapy exercises for	EG:at home ,<br>CG:local health district	NR	EG:<br>(4 weeks)<br>5 sessions / week.	NR	UL	3D motion tracking system / screen	upper limb function (FMA-UE)	NR

Study author & year (country)	design	Participants' number (age)	Time from a stroke in days(stage)	Stroke subtype in %	Cognitive state	Intervention	Setting of treatment	Revision of treatment plan	Dose of exercises	multimorbid conditions (event/total)	Target region	Devices used to deliver VR exercises	Primary Outcomes (Outcome measure scale)	Adverse effects
					lower than 62 points at the De Renzi Test) was excluded.	the upper limb with a strategy of progressive complexity.			(60 min /session) CG: (4 weeks) 5 sessions / week. (60 min /session)					
Llore´ns ., 2015 (Spain)	RCT	Total=30 E:15 (55.47± 9.63) C:15 (55.6± 7.29)	E:334.13±60.79 C:316.73±49.81 (chronic)	E: ischemic 60% Haemorrhagic 40% C: ischemic 66.7% Haemorrhagic 33.3%	Mini-Mental State Examination score >23	EG:The virtual environment used in the experiment represented the participants' feet and their movements in an empty scenario, which consisted of a checkered floor that facilitated the perception of depth, with a central circle that represented the center of the virtual environment., (unsupervised) CG:The virtual environment used in the	EG:at home , CG:at clinic	weekly	EG : (20 sessions) 3 sessions / week. (45 min / session). CG: (20 sessions) 3 sessions / week. (45 min / session).	NR	Trunk, LL	television, conventional computer or labtop, Microsoft Kinect	Balance (BBS)	NR

Study author & year (country)	design	Participants' number (age)	Time from a stroke in days(stage)	Stroke subtype in %	Cognitive state	Intervention	Setting of treatment	Revision of treatment plan	Dose of exercises	multimorbid conditions (event/total)	Target region	Devices used to deliver VR exercises	Primary Outcomes (Outcome measure scale)	Adverse effects
						experiment represented the participants' feet and their movements in an empty scenario, which consisted of a checkered floor that facilitated the perception of depth, with a central circle that represented the center of the virtual environment.								
Zondervan ., 2016 (USA)	RCT crossover	Total=17<br>E:9 (60)<br>C:8(59)	E:1945±1511<br>C:1157±605<br>(chronic)	NM	Patients are excluded if they have apraxia.	EG:MusicGlove therapy (unsupervised exercises)<br>CG:conventional therapy group using a booklet of tabletop exercises.	EG:at home ,<br>CG:at home	weekly	EG:<br>(3 weeks)<br>3 sessions / week.<br>(60 min / session).<br>CG:<br>(3 weeks)<br>3 sessions / week.<br>(60 min / session).	NR	UL, hand	MusicGlove device and a laptop	Upper limb function (BBT)	NR

Study author & year (country)	design	Participants' number (age)	Time from a stroke in days(stage)	Stroke subtype in %	Cognitive state	Intervention	Setting of treatment	Revision of treatment plan	Dose of exercises	multimorbid conditions (event/total)	Target region	Devices used to deliver VR exercises	Primary Outcomes (Outcome measure scale)	Adverse effects
Hernandez ., 2022 (canada)	RCT	Total=51<br>E:26 (59.8± 13.1)<br>C:25 (56.7± 11.2)	E: median (IQR) 5.3 (1.5-8.1)<br>C: median (IQR) 4.4 (2.2-7.4)<br>(chronic)	E: ischemic 54%<br>Haemorrhagic 27%<br>Unknown 19%<br>C: ischemic 40%<br>Haemorrhagic 28%<br>Unknown 32%	Patients are excluded if they have difficulty in understanding simple verbal instructions.	EG:home-based exercise program via the Jintronix system monitored offline by a therapist for upper limb exercises. CG:home-based exercise program manual [GRASP] provided by a therapist without further supervision.	EG:at home , CG:at home	twice/ week	EG:5 sessions / week (≥20 min / session) CG: Participant was encouraged to engage in the program as many times a week as possible.	NR	UL	computer, large screen , Microsoft Kinect .	Upper limb function (FMA-UE)	NR
	ARAT:action research arm test.BBS:balance berg scale.CAHAI: Chedoke Arm and Hand Activity Inventory.CG:control group.EG:experimental group.FAC:functional ambulation category.<br>FES-I:fall efficacy scale -international.GRASP : Graded Repetitive Arm Supplementary Program.HEP:home-based exercise program.LL:lower limb.NR : not reported.POMA:performance oriented mobility assessment.RCT:randomised controlled trial.TIA:transient ischemic attack.TUG:time up and go test.UL:upper limb.VR:virtual reality.													

Table S6:Summary of quantitative outcomes .

Study	Motor outcomes													
	Upper limb and hand function		Lower limb function		Muscle strength		Mobility		balance		Gait		Fall	
	W	B	W	B	W	B	W	B	W	B	W	B	W	B
Cramer ., 2019	(FMA UE)<br>↑ + *<br>(Box and blocks test)	(FMA UE)<br>↑ +<br>(Box and blocks test)												

	↑ + * (1 month)<br>NR (follow-up)<br>(Stroke Impact Scale hand motor domain scores)<br>↑ + *	↑ + (1 month)<br>NR (follow-up)<br>(Stroke Impact Scale hand motor domain scores)<br>↑ + ?												
Yang ., 2022	(FMA UE)<br>↑ + *	(FMA UE)<br>↑ +<br>P=0.76	(FMA LE)<br>↑ + *	(FMA LE)<br>↑ +<br>P=0.843					(BBS)<br>↑	(BBS)<br>P=0.452	(FAC)<br>↑	(FAC)<br>P=0.122		
Adie ., 2017	ARAT<br>↑ + *	ARAT<br>↑ +<br>p = 0.12												
Ballester ., 2017	(FMA UE)<br>↑ (week 3)<br>↓ (week 12)<br>(CAHAI)<br>↑+ * (week 3)<br>↓ ? (week 12)	(FMA UE)<br>P=0.33 (week 3)<br>P=0.21 (week 12)<br>(CAHAI)<br>P=0.05 * (week 3)<br>P=0.61 (week 12)			(MRC-p)<br>↑ (3 week)<br>↓ (12 week)<br>(MRC-d)<br>↑ (3 week)<br>↑ (12 week)	(MRC-p)<br>P=0.61(3 week)<br>P=0.06 (12 week)<br>(MRC-d)<br>P=0.74<br>P=0.98								
Lee ., 2022							(TUG)<br>↓ + * (post)<br>↓ + * (follow up)	(TUG)<br>↓ + * (post)<br>p=0.016<br>↓ + (follow up)	(BBS)<br>↑+* (post)<br>↑+* (follow up)	(BBS)<br>↑ +(post)<br>↑+(follow up)			(FES-I)<br>↓+ * (post)<br>↓+ * (follow up)	(FES-I)<br>↓+ * (post)<br>↓+ * (follow up)

							(POMA)<br>↑ (post)<br>↑ (follow up)	(POMA)<br>? (post)<br>? (Follow up)						
Piron ., 2009	(FMA UE)<br>↑ + *(initial assessment)<br>↑ + * (follow up)	(FMA UE)<br>↑ + *(initial assessment)<br>↑ + * (follow up)												
Llore´ns ., 2015									(BBS)<br>↑+* (post)<br>↑+* (follow up)<br>(POMA-B)<br>↑+* (post)<br>↑+* (follow up)<br>(BBA)<br>↑+* (post)<br>↑+* (follow up)	(BBS)<br>↑ +(post)<br>↑+(follow up)<br>(POMA-B)<br>↑ +(post)<br>↑+(follow up)<br>(BBA)<br>↑ +<br>(post)<br>↑+<br>(follow up)	(POMA-G)<br>↑+* (post)<br>↑+* (follow up)	(POMA-G)<br>↑ +(post)<br>↑+(follow up)		
Zondervan ., 2016	(ARAT)<br>↑ + (initial analysis)<br>↑ + * (crossover analysis)	(ARAT)<br>↑ + (initial analysis)<br>P=0.29												

	(Nine hole pig test)<br>↑ + (initial analysis)<br><br>(Box and blocks test)<br>↑ + ? (initial analysis)<br><br>Motor Activity Log<br>(quality of movement)<br>↑ + ? (initial analysis)<br><br>Motor Activity Log<br>(amount of use)<br>↑ + ? (initial analysis)	↑ + * (crossover<br>analysis)<br>(Nine hole pig test)<br>↑ + (initial analysis)<br>P=0.33<br>(Box and blocks test)<br>↑ + (initial analysis)<br>P=0.25<br>Motor Activity Log<br>(quality of movement)<br>↑ + *(initial analysis)<br>P=0.007<br>Motor Activity Log<br>(amount of use)<br>↑ + *(initial analysis)<br>P=0.04												
Hernandez ., 2022	(FMA UE)<br>↑ + *	(FMA UE)<br>↑ + *												

W= within intervention group

B=between intervention and control group

↑= increase value within the intervention group

↓=decrease value within the interventional group

+ = positive change (improvement)

- = negative change (no improvement)

*Significant difference

? significance not reported.

NR= not reported

Table S7 (summary of the quantitative outcome effect measures)

Study		Upper limb function																															
		FM motor score UE				Action research arm test				Box and Blocks Test				Stroke Impact Scale hand motor domain scores				Nine Hole Peg Test				CAHAI				MAL (quality of movement)				MAL (amount of use)			
		Experimental		Control		Experimental		Control		Experimental		Control		Experimental		Control		Experimental		Control		Experimental		Control		Experimental		Control		Experimental		Control	
		Mean difference	SD	Mean difference	SD	Mean difference	SD	Mean difference	SD	Mean difference	SD	Mean difference	SD	Mean difference	SD	Mean difference	SD	Mean difference	SD	Mean difference	SD	Mean difference	SD	Mean difference	SD	Mean difference	SD	Mean difference	SD	Mean difference	SD	Mean difference	SD
**Cramer ., 2019**	baseline to 30 days	7.86	6.68	8.36	7.04					9.5	NR	8.8	NR	23.7	NR	29.2	NR																
**Yang ., 2022**	baseline-post	2.04	4.57	2.34	3.98																												
**Adie., 2017**	baseline-post (6 weeks)					6.28	9.22	7.63	9.36																								
	baseline-post (6 months)					7.49	10.54	8.73	12.59																								
**Ballester ., 2017**	baseline-post (week 3)	0.35	1.62	1.22	3.84																	1.53	2.4	-0.67	6.01								
	baseline-post (week 12)	-0.18	3.5	1.39	3.63																	-0.06	6.51	0.44	5.46								
**Piron ., 2009**	T30-T60	**T30 = 48.5* (7.8), T60 = 53.6* (7.7). results are in mean (SD).**		**T30=47.3 (4.6) , 49.5 (4.8). results are in mean (SD).**																													
	T60-T90	**T60 = 53.6 (7.7) , T90 = 53.1 (7.3). results are in mean (SD).**		**T60=49.5 (4.8) , T90=48.8 (5.1). results are in mean (SD).**																													
**Zondervan ., 2016**	baseline-1 month post treatment					0.7	2.3	-0.6	2.9	2.3	6.2	4.3	5					1.3	6.9	-0.2	6					0.82	0.48	0.09	0.58	0.86	0.64	0.26	0.69
**Hernandez ., 2022**	NO DATA AVAILABLE	NR		NR																													

All results presented in mean difference (SD) except results with [*].
NR = not reported

Study		Lower limb function				Muscle strength								Mobility							
		FM motor score LE				MRC proximal				MRC distal				TUG				POMA			
		Experimental		Control		Experimental		Control		Experimental		Control		Experimental		Control		Experimental		Control	
		Mean difference	SD	Mean difference	SD	Mean difference	SD	Mean difference	SD	Mean difference	SD	Mean difference	SD	Mean difference	SD	Mean difference	SD	Mean difference	SD	Mean difference	SD
**Cramer ., 2019**	baseline to 30 days									9.5	NR	8.8	NR	23.7	NR	29.2	NR				
**Yang ., 2022**	baseline-post	1.84	3.45	1.63	4.85																
**Ballester ., 2017**	baseline-post (week 3)					0.06	0.24	0.11	0.32	0.06	0.43	0.11	0.47								
	baseline-post (week 12)					-0.12	0.78	0.28	0.46	0.29	0.77	0.17	0.62								
**Lee ., 2022**	baseline - post (4 weeks)													2.3	***[95% (CI) 1.22, 3.38]**	0.91	***[95% CI 0.13, 1.68]**	-0.9	NM	-0.7	NM
	baseline -follow up (4 weeks)													2.47	***[95% CI 1.39, 3.54]**	1.61	***[95% CI 0.82, 2.40]**	-1.2	NM	-0.9	NM

All results presented in mean difference (SD) except results with [*].
NR = not reported
CI = confidence interval

Study		Balance								Gait								Fall			
		POMA-B				BBS				POMA-G				FAC				FES-I			
		Experimental		Control		Experimental		Control		Experimental		Control		Experimental		Control		Experimental		Control	
		Mean difference	SD	Mean difference	SD	Mean difference	SD	Mean difference	SD	Mean difference	SD	Mean difference	SD	Mean difference	SD	Mean difference	SD	Mean difference	SD	Mean difference	SD
**Yang ., 2022**	baseline-post					1.88	5.09	1.13	3.69					0.26	0.83	0.08	0.6				
**Lee ., 2022**	baseline - post (4 weeks)					-2.66	***[95% CI -4.38, -0.94]**	-1.67	***[95% CI -2.88, -0.45]**									-3.4	NR	0.4	NR
	baseline -follow up (4 weeks)					-3.17	***[95% CI -5.10, -1.24]**	-2.41	***[95% CI -3.66, -1.16]**									-5.1	NR	-0.3	NR
**Llore´ns ., 2015**	base line to week 8	0.86	1.5	0.26	0.45	3.66	2.38	2.26	1.76	0.93	0.59	0.4	0.6								
	week 8 to week 12	0.67	0.59	0.2	0.41	0.33	0.61	0.67	0.17	0.07	0.45	0.13	0.3								

All results presented in mean difference (SD) except results with [*].
NR = not reported
CI = confidence interval

Table S8:Summary of qualitative studies characteristics .

Study author & year (country)	Objectives	design	Participants' number (age)	Gender	Stage of stroke	Intervention	Setting of treatment	Inclusion criteria	Exclusion criteria	Mode of delivery of VR	Target region	Outcomes	Underlying theory	Methods of data collection	Methods of data analysis
Dorra Rakia Allegue., 2022 (Canada)	To explore the indicators of empowerment among stroke survivors, and investigate the determinants of VirTele use among stroke survivors'	(qualitative) multiple case study	3 patients Mean ± sd (58.8 ±19.4)	1 male 2 female	Chronic	VirTele exercises using the Jintronix exergame for UE training	At home	First-time unilateral ischemic or hemorrhagic stroke or no residual deficits from a previous stroke.<br>Patient can move the game avatar with impaired limb).	Medically unstable (eg, uncontrolled cardiac condition).<br>Severe cognitive or communication deficits.<br>Visual impairments limiting the ability to use the games.<br>UE mobility restrictions limiting the ability to play (eg, restricted shoulder movements because of pain)'	non-immersive	UE	1- indicators of empowerment among stroke survivors.<br>2- determinants of VirTele use among stroke survivors.	UTAUT (The Unified Theory of Acceptance and Use of Technology).<br>SDT (self-determination theory)	semi structured interviews (lasting from 30 minutes to 1 hour)	thematic analysis
Wingham.,2015 (UK)	To understand stroke survivors and their caregivers' experience and acceptability of using the Nintendo Wii SportsTM	(qualitative)	18 patients- 10 caregiver<br>Age of participants : median (min–max age) 65 (35–84)	11 male 7 female	Subacute-chronic	Wii games included bowling, tennis, golf, boxing and baseball	At home	Patient is able to take part in semistructured interview.<br>Aged 18 or over and could give informed consent.	NR	Semi-immersive	UE	experience and acceptability of using the Wii games in stroke telerehabilitation.	no underlying theory reported	Semi-structured interviews	thematic analysis

Study author & year (country)	Objectives	design	Participants' number (age)	Gender	Stage of stroke	Intervention	Setting of treatment	Inclusion criteria	Exclusion criteria	Mode of delivery of VR	Target region	Outcomes	Underlying theory	Methods of data collection	Methods of data analysis
	games (WiiTM) as a home-based arm rehabilitation tool														
Standen ., 2014 (UK)	To determine patients experience of using the glove and barriers to using it in the recommended way and to the recommended levels	(qualitative)	11 patients (NR)	5 male 6 female	Subacute-chronic	virtual glove and games (Spacerace required pronation and supination of the hand to guide a spacecraft through obstacles. Spongeball required the user to open his or her fist and extend the fingers in order to release a ball to hit a target. Balloonpop required a balloon to be grasped and popped by moving it to a pin protruding from the floor)	At home	aged 18 years or older, had a confirmed diagnosis of stroke, were no longer receiving any other intensive rehabilitation (intermediate care, early supported discharge), and still had residual upper limb dysfunction.	NR	Semi-immersive	Hand	experience of using the glove	no underlying theory reported	Semi-structured interviews	thematic analysis
Chen ., 2020 (USA)	To explore user acceptance of a homebased stroke telerehabilitation system	(qualitative)	13 patients mean±sd (70.61±11.14)	11 male 2 female	subacute	The system delivers treatment sessions in the form of daily guided rehabilitation games, exercises for UL , and stroke education in the patient's home.	At home	1.Age ≥18 years at the time of randomization 2.Stroke that is radiologically verified, due to ischemia or to intracerebral hemorrhage,	1.A major, active, coexistent neurological or psychiatric disease, including alcoholism or dementia	non-immersive	UE	user acceptance of the telerehabilitation system	UTAUT (The Unified Theory of Acceptance and Use of Technology)	Semi-structured interviews	thematic analysis

Study author & year (country)	Objectives	design	Participants' number (age)	Gender	Stage of stroke	Intervention	Setting of treatment	Inclusion criteria	Exclusion criteria	Mode of delivery of VR	Target region	Outcomes	Underlying theory	Methods of data collection	Methods of data analysis
								and with time of stroke onset 4–36 weeks prior to randomization<br>3.Arm motor FM score of 22–56 (out of 66, higher is better) at the Screening Visit<br>4.Box & Block Test score with affected arm is at least 3 blocks in 60 seconds at the Screening Visit<br>5.Informed consent signed by the subject<br>6.Behavioral contract signed by the subject	2.A diagnosis (apart from the index stroke) that substantially affects paretic arm function<br>3.A major medical disorder that substantially reduces the likelihood that a subject will be able to comply with all study procedures<br>4.Severe depression, defined as Geriatric Depression Scale Score >10<br>5.Significant cognitive impairment, defined as Montreal Cognitive Assessment score < 22<br>6.Deficits in communication that interfere with reasonable study participation<br>7.A new symptomatic stroke has occurred since						

Study author & year (country)	Objectives	design	Participants' number (age)	Gender	Stage of stroke	Intervention	Setting of treatment	Inclusion criteria	Exclusion criteria	Mode of delivery of VR	Target region	Outcomes	Underlying theory	Methods of data collection	Methods of data analysis
									the index stroke that occurred 4–36 weeks prior to randomization<br>8.Lacking visual acuity, with or without corrective lens, of 20/40 or better in at least one eye<br>9.Life expectancy < 6 months<br>10.Pregnant<br>11.Receipt of Botox to arms, legs, or trunk in the preceding 6 months, or expectation that Botox will be administered to the arm, leg, or trunk prior to completion of the 30 Day Follow-Up Visit<br>12.Unable to successfully perform all three of the rehabilitation exercise test examples						

Study author & year (country)	Objectives	design	Participants' number (age)	Gender	Stage of stroke	Intervention	Setting of treatment	Inclusion criteria	Exclusion criteria	Mode of delivery of VR	Target region	Outcomes	Underlying theory	Methods of data collection	Methods of data analysis
									13.Unable or unwilling to perform study procedures/therapy, or expectation of noncompliance with study procedures/therapy<br>14.Concurrent enrollment in another investigational study<br>15.Non-English speaking, such that subject does not speak sufficient English to comply with study procedures<br>16.Expectation that subject cannot participate in study visits<br>17.Expectation that subject will not have a single domicile address during the six weeks of therapy,						

Study author & year (country)	Objectives	design	Participants' number (age)	Gender	Stage of stroke	Intervention	Setting of treatment	Inclusion criteria	Exclusion criteria	Mode of delivery of VR	Target region	Outcomes	Underlying theory	Methods of data collection	Methods of data analysis
									within 25 miles of the central study site and with Verizon wireless reception.						

References:

67. Pollock, A., et al., *Interventions for improving upper limb function after stroke.* Cochrane Database Syst Rev, 2014. **2014**(11): p. Cd010820.
68. Frenkel-Toledo, S., et al., *Stroke Lesion Impact on Lower Limb Function.* Front Hum Neurosci, 2021. **15**: p. 592975.
69. Pollock, A.S., et al., *What is balance?* Clin Rehabil, 2000. **14**(4): p. 402-6.
70. Suchomel, T.J., et al., *The Importance of Muscular Strength: Training Considerations.* Sports Med, 2018. **48**(4): p. 765-785.
71. Barbosa, H., et al., *Human mobility: Models and applications.* Physics Reports, 2018. **734**: p. 1-74.
72. Montero-Odasso, M., et al., *World guidelines for falls prevention and management for older adults: a global initiative.* Age and Ageing, 2022. **51**(9).
73. Mirelman, A., et al., *Gait.* Handb Clin Neurol, 2018. **159**: p. 119-134.
74. Oben, P., *Understanding the Patient Experience: A Conceptual Framework.* Journal of Patient Experience, 2020. **7**(6): p. 906-910.
